# Use of Proton-Pump Inhibitors Predicts Heart Failure and Death in Patients with Coronary Artery Disease

**DOI:** 10.1371/journal.pone.0169826

**Published:** 2017-01-19

**Authors:** Ana María Pello Lázaro, Carmen Cristóbal, Juan Antonio Franco-Peláez, Nieves Tarín, Álvaro Aceña, Rocío Carda, Ana Huelmos, María Luisa Martín-Mariscal, Jesús Fuentes-Antras, Juan Martínez-Millá, Joaquín Alonso, Óscar Lorenzo, Jesús Egido, Lorenzo López-Bescós, José Tuñón

**Affiliations:** 1 Department of Cardiology, IIS-Fundación Jiménez Díaz, Madrid, Spain; 2 Department of Cardiology, Hospital de Fuenlabrada, Madrid, Spain; 3 Rey Juan Carlos University, Alcorcón, Madrid, Spain; 4 Department of Cardiology, Hospital Universitario de Móstoles, Madrid, Spain; 5 Department of Cardiology, Hospital Universitario Fundación Alcorcón, Madrid, Spain; 6 Department of Cardiology, Hospital de Getafe, Madrid, Spain; 7 Autónoma University, Madrid, Spain; 8 Laboratory of Vascular Pathology, IIS-Fundación Jiménez Díaz, Madrid, Spain; 9 CIBERDEM, Madrid, Spain; University of Bologna, ITALY

## Abstract

**Objectives:**

Proton-pump inhibitors (PPIs) seem to increase the incidence of cardiovascular events in patients with coronary artery disease (CAD), mainly in those using clopidogrel. We analysed the impact of PPIs on the prognosis of patients with stable CAD.

**Methods:**

We followed 706 patients with CAD. Primary outcome was the combination of secondary outcomes. Secondary outcomes were 1) acute ischaemic events (any acute coronary syndrome, stroke, or transient ischaemic attack) and 2) heart failure (HF) or death.

**Results:**

Patients on PPIs were older [62.0 (53.0–73.0) vs. 58.0 (50.0–70.0) years; p = 0.003] and had a more frequent history of stroke (4.9% vs. 1.1%; p = 0.004) than those from the non-PPI group, and presented no differences in any other clinical variable, including cardiovascular risk factors, ejection fraction, and therapy with aspirin and clopidogrel. Follow-up was 2.2±0.99 years. Seventy-eight patients met the primary outcome, 53 developed acute ischaemic events, and 33 HF or death. PPI use was an independent predictor of the primary outcome [hazard ratio (HR) = 2.281 (1.244–4.183); p = 0.008], along with hypertension, body-mass index, glomerular filtration rate, atrial fibrillation, and nitrate use. PPI use was also an independent predictor of HF/death [HR = 5.713 (1.628–20.043); p = 0.007], but not of acute ischaemic events. A propensity score showed similar results.

**Conclusions:**

In patients with CAD, PPI use is independently associated with an increased incidence of HF and death but not with a high rate of acute ischaemic events. Further studies are needed to confirm these findings.

## Introduction

The efficacy of proton-pump inhibitors (PPIs) in suppressing gastric acid secretion has led them to be preferred over other drugs such as histamine H_2_ receptor antagonists [1].

In patients with coronary artery disease (CAD), aspirin is used to decrease the incidence of cardiovascular events, and in patients who have undergone stent placement or have suffered an acute coronary syndrome, a P_2_Y_12_ receptor blocker such as clopidogrel is added. These antiplatelet agents, however, may favour the development of gastrointestinal (GI) complications. Prolonged aspirin therapy is associated with GI ulceration and bleeding, which have been attributed to mucosal injury caused by inhibition of prostaglandin and to systemic inhibition of thromboxane A_2_ production, respectively. In addition, clopidogrel may impair the healing of gastric erosions, exacerbating GI complications associated with the concomitant administration of aspirin [2].

PPIs are indicated in CAD patients to decrease the risk of upper GI haemorrhage due to antiplatelet therapy [3]. However, patients treated with PPIs may develop osteoporosis-related fractures [4], pneumonia, *Clostridium difficile* infection, acute interstitial nephritis, and micronutrient deficiencies [5,6]. In addition, it has been suggested that PPIs may increase the incidence of cardiovascular events in CAD patients by decreasing the effect of aspirin—and, mainly, clopidogrel—on platelet aggregation [7–11]. Although several pharmacodynamic studies have suggested an interaction between PPIs and antiplatelet drugs [12], clinical studies have shown divergent results [13,14].

In this study we assessed the potential association between the use of PPIs and adverse outcome in patients with stable CAD who had developed an acute coronary syndrome 6–12 months before.

## Materials and Methods

### Patients

The research protocol complies with the Declaration of Helsinki and was approved by the ethics committees of the participating hospitals. All patients included in the study signed informed consent documents. As described in detail previously, the BACS & BAMI (Biomarkers in Acute Coronary Syndrome & Biomarkers in Acute Myocardial Infarction) studies included patients admitted to 4 hospitals in Madrid with either non-ST elevation acute coronary syndrome (NSTEACS) or ST elevation myocardial infarction (STEMI) [15]. Detailed inclusion and exclusion criteria have been previously reported [15,16]. Patients were seen on an outpatient basis 6 months after initial diagnosis. At this time plasma was withdrawn and a complete set of clinical variables was recorded. At this outpatient visit we started a prospective follow-up relating the clinical and analytical findings obtained with the outcome of the patients.

Between July 2006 and April 2010, 1,898 patients were discharged from the study hospitals with a diagnosis of NSTEACS or STEMI [15]. Of these, 838 were eventually included in the study [15]. The remaining patients were not included based on the following exclusion criteria, that have been described previously [15,16]: age over 85 years (17.3%), disorders limiting survival (29.0%), impossibility to perform cardiac revascularisation (14.5%), coexistence of other significant cardiac disorders (6.8%), impossibility to perform follow-up (12.0%), clinical instability beyond the sixth day at the index event (9.1%), refusal to participate in the study (2.0%), and impossibility of the investigators to include them (9.3%). Of the 838 patients included during the acute event, 711 attended the outpatient visit at 6 months and had adequate plasma samples stored. This visit took place between January 2007 and February 2011. Final follow-up visits took place in May 2012. Five patients were lost to follow-up, leaving a total of 706 patients for analysis.

### Study Design

As explained previously, at baseline, clinical variables were recorded and twelve-hour fasting venous blood samples were withdrawn and collected in EDTA. Blood samples were centrifuged at 2,500 g for 10 minutes and plasma was stored at –80°C. Patients were seen every year at their hospital. At the end of follow-up (maximum 4.6 years) medical records were reviewed and patient status was confirmed by telephone contact.

The primary outcome was the composite of the secondary end points. The secondary outcomes were 1) recurrence of acute ischaemic events such as NSTEACS, STEMI, stroke, or transient ischaemic attack, and 2) incidence of heart failure (HF) or death from any cause. NSTEACS was defined as rest angina lasting more than 20 minutes in the previous 24 hours or new-onset class III-IV angina, along with transient ST-segment depression or T-wave inversion in the electrocardiogram considered diagnostic by the attending cardiologist and/or troponin elevation. STEMI was defined as symptoms compatible with angina lasting more than 20 minutes and ST elevation in 2 adjacent leads in the electrocardiogram without response to nitroglycerin, and troponin elevation. Stroke was defined as rapid onset of a persistent neurologic deficit attributable to a focal vascular cause and lasting more than 24 hours, supported in most cases by imaging studies. A transient ischaemic attack was defined as a stroke with signs and symptoms resolving before 24 hours without acute ischaemic lesions as assessed by imaging studies. The HF end point was assigned to patients hospitalised for this reason. Events were adjudicated by 2 investigators. Cerebrovascular events were adjudicated with the assistance of a neurologist.

### Analytical Studies

Laboratory analyses were carried out in the Clinical Biochemistry Laboratory of the IIS-Fundación Jiménez Díaz by investigators who were unaware of the clinical data. As in previous papers studying this population, high-sensitivity C-reactive protein was determined by latex-enhanced immunoturbidimetry (ADVIA 2400 Chemistry System, Siemens, Germany) [15]. Lipid, glucose, and creatinine determinations were carried out by standard methods (ADVIA 2400 Chemistry System, Siemens, Germany) [15].

### Statistical Analysis

Quantitative data following a normal distribution are presented as mean ± standard deviation and compared using the Student “t” test. Data that were not normally distributed are displayed as median (interquartile range) and compared using the Mann-Whitney test. Qualitative variables are displayed as percentages and were compared by χ^2^ or Fisher exact test when appropriate. Kaplan-Meier curves and the log-rank test were used to compare time to outcome according to therapy with PPIs. Cox proportional hazards modelling was used with forward stepwise selection to assess the variables associated with the primary and secondary outcomes. In model 1, clinical and analytical variables were studied: age, sex, diabetes, smoking status, hypertension, body-mass index, LDL, HDL, triglycerides, previous history of peripheral artery disease, cerebrovascular events, atrial fibrillation or coronary artery by-pass graft; ejection fraction<40%, glomerular filtration rate assessed as Chronic Kidney Disease Epidemiology Collaboration method (CKD-EPI), high-sensitivity C-reactive protein, type of last acute coronary event, number of diseased vessels, percutaneous or surgical revascularisation, use of drug-eluting stents and existence of complete revascularisation at that event. In model 2, treatment with PPIs and other therapies were added: aspirin, clopidogrel, statins, acenocumarol, angiotensin-converting enzyme inhibitors, angiotensin receptor blockers, betablockers, nitrates/nitroglycerin and diuretics. We considered significant when “*p*” value was lower than 0.05 (two-tailed). Analyses were carried out with SPSS 19.0 (SPSS Inc., New York).

A propensity score adjustment was performed using a multivariable logistic regression model in 2 steps, including clinical variables in the first one and adding the therapies used in the second step.

### Summary of the Study

In conclusion, in this study we have followed 706 patients with chronic CAD, assessing the potential relationship of PPI use with the development of acute ischaemic events, heart failure (HF), or death.

## Results

Mean follow-up was 2.2±0.99 years. Time from the previous acute coronary event was 7.5 ± 3.0 months.

Of the 706 patients analysed, 431 (61.04%) were receiving PPIs. Most of them (405 patients, (57.4%)) were taking omeprazole, 19 (2.7%) pantoprazole, 6 (0.8%) lansoprazole, and 1 patient (0.1%) was taking rabeprazole. There were 72 (10.2%) patients treated with histamine H_2_ receptor antagonists, and the remaining patients did not receive gastric protectors. Patients receiving these drugs were older and had a more frequent history of cerebrovascular events than those not receiving them. No differences were observed in cardiovascular therapy or any other clinical or analytical variables (Table 1).

**Table 1:** Characteristics of patients with and without treatment with proton-pump inhibitors.

	Patients receiving PPIs (N = 431)	Patients not receiving PPIs (N = 275)	P Value
Age, y	62.0 (53.0–73.0)	58.0 (50.0–70.0)	**0.003**
Male sex (%)	74.5	76.4	0.593
Caucasian (%)	97.9	95.6	0.110
Body-mass index (Kg/m^2^)	28.7±4.4	28.8±4.7	0.861
Diabetes (%)	22.7	24.0	0.715
Present smoker (%)	5.6	8.0	0.213
Hypertension (%)	66.8	62.5	0.257
Peripheral artery disease (%)	3.9	3.6	1.000
Cerebrovascular events (%)	4.9	1.1	**0.004**
Previous CABG (%)	9.3	6.9	0.329
Atrial fibrillation (%)	3.5	1.6	0.381
Ejection fraction < 40% (%)	11.8	12.0	0.725
**MEDICAL THERAPY**			
Aspirin (%)	91.4	92.7	0.573
Clopidogrel (%)	66.8	69.1	0.564
Acenocoumarol (%)	6.7	5.1	0.423
Statins (%)	87.5	87.6	1.000
ACEI (%)	55.9	54.2	0.698
ARB (%)	17.4	14.9	0.406
Betablockers (%)	74.2	79.6	0.103
Nitrates/Nitroglycerin (%)	17.2	16.7	0.918
Diuretics (%)	21.8	16.0	0.064
**DATA FROM LAST ACUTE CORONARY EVENT**			
STEMI/NSTEACS (%)	39.9/60.1	36.7/63.3	0.428
Number of vessels diseased	1.39±0.79	1.35±0.83	0.517
Complete revascularisation (%)	6.3	4.7	0.410
Drug-eluting stent (%)	44.5	50.9	0.105
PCI (%)	74.0	73.5	0.930
CABG (%)	6.3	4.7	0.384
**ANALYTICAL DATA**			
LDL cholesterol (mg/dl)	84.3±26.4	81.4±24.3	0.149
HDL cholesterol (mg/dl)	43.7±10.6	44.1±11.4	0.683
Triglycerides (mg/dl)	129.7±71.3	132.7±98.3	0.640
GFR (ml/min/1.73 m^2^)	75.7±21.7	76.5±18.6	0.600
HS C-reactive protein (mg/L)	4.66±10.39	4.24±8.38	0.569

**ACEI:** angiotensin-converting enzyme inhibitors; **ACS:** acute coronary syndrome; **ARB:** angiotensin receptor blockers; **CABG:** coronary artery by-pass graft; **GFR:** glomerular filtration rate (Chronic Kidney Disease Epidemiology Collaboration method); **HDL:** high-density lipoprotein; **HS:** high-sensitivity; **LDL:** low-density lipoprotein; **NSTEACS:** Non-ST elevation acute coronary syndrome; **PCI:** percutaneous coronary intervention; **PPIs:** Proton-Pump Inhibitors; **STEMI:** ST-elevation myocardial infarction

Seventy-eight patients developed the primary outcome of acute ischaemic events, HF, or death. Sixty-three (14.61%) patients taking PPIs and 15 (5.45%) not on PPIs met this outcome. Twelve patients developed 2 events, 5 patients experienced 3 events and, the remainder, one event with a total of 100 events. At multivariable analysis we included the variables displayed in Table 1 in two models, as described in the Methods section. PPI use was an independent predictor of the primary outcome, along with hypertension, age, body-mass index, estimated glomerular filtration rate, atrial fibrillation, and nitrate use (Table 2). The interaction between PPIs and clopidogrel did not reach statistical significance (p = 0.463).

**Table 2:** Cox proportional hazards model for the incidence of primary outcome: acute ischaemic events, heart failure, or death.

	Model 1				Model 2			
	Hazard Ratio	95% CI		*P* value	Hazard Ratio	95% CI		*P* value
		Lower	Upper			Lower	Upper	
**Hypertension**	2.271	1.106	4.665	0.025	2.529	1.232	5.195	0.011
**Atrial fibrillation**	2.102	1.111	3.977	0.022	2.598	1.390	4.854	0.003
**Age**^a^	1.036	1.008	1.064	0.011	---	---	---	---
**BMI**^b^	1.074	1.020	1.131	0.007	1.060	1.006	1.116	0.029
**CKD-EPI**^c^	0.982	0.967	0.997	0.021	0.974	0.962	0.987	<0.001
**Nitrates**	---	---	---	---	2.669	1.626	4.378	<0.001
**PPIs**	---	---	---	---	2.281	1.244	4.183	0.008

Model 1: Risk adjusted for age, sex, diabetes, smoking status, hypertension, body-mass index, low-density lipoprotein, high-density lipoprotein, and triglyceride plasma levels; previous history of peripheral artery disease, cerebrovascular events, atrial fibrillation or coronary artery by-pass graft; ejection fraction <40%, glomerular filtration rate assessed as Chronic Kidney Disease Epidemiology Collaboration method, high-sensitivity C-reactive protein; type of last acute coronary event, number of diseased vessels, percutaneous or surgical revascularisation, use of drug-eluting stents and existence of complete revascularisation at that event.

Model 2: Risk adjusted for factors in model 1 plus therapy with proton-pump inhibitors, aspirin, clopidogrel, statins, acenocoumarol, angiotensin-converting enzyme inhibitors, angiotensin receptor blockers, betablockers, diuretics and nitrates/nitroglycerin.

**BMI:** body-mass index; **CI:** confidence interval; **CKD-EPI:** glomerular filtration rate assessed according to the Chronic Kidney Disease Epidemiology Collaboration method; **PPIs:** proton-pump inhibitors.

^a^: hazard ratio estimated by every increase of 1 year.

^b^: hazard ratio estimated by every increase of 1 kg/m^2^.

^c^: hazard ratio estimated by every increase of 1 ml/min/1.73m^2^

When multivariable Cox regression analysis was performed comparing patients with omeprazol with those no taking this drug (then including 26 patients using other PPIs in this group) omeprazol was an independent risk predictor (HR 2.343 [1.320–4.158]; p = 0.004) along with hypertension (HR 2.591 [1.262–5.321]; p = 0.010), body-mass index (HR 1.060 [1.006–1.116]; p = 0.028), atrial fibrillation (HR 2.561 [1.372–4.781]; p = 0.003), glomerular filtration rate (HR 0.975 [0.963–0.987]; p<0.001), and use of nitrates (HR 2.678 [1.626–4.404]; p<0.001). Comparing patients with omeprazol with the remaining cases not on PPIs confirmed that omeprazol (HR 2.371 [1.290–4.358]; p = 0.005) was an independent predictor of the primary outcome along with the same other described variables. Given that the number of cases on anti-H2 and without gastric protectors was very low it was not possible to perform other comparisons.

Fifty-three patients developed acute ischaemic events. Forty-two (9.74%) of the patients on PPIs and 11 (4%) of those not receiving these drugs met this end point. There were 4 episodes of STEMI, 22 of NSTEACS, 17 of unstable angina, 8 strokes, and 10 transient ischaemic attacks. Four patients experienced 2 events and 2 patients experienced 3 events. By multivariable analysis, age, body-mass index, and treatment with nitrates, but not with PPIs, were independent predictors of this end point (Table 3).

**Table 3:** Cox proportional hazards model for the incidence of acute ischaemic events.

	Model 1				Model 2			
	Hazard ratio	95% CI		*P* value	Hazard ratio	95% CI		*P* value
		Lower	Upper			Lower	Upper	
**Age**^a^	1.045	1.015	1.065	<0.001	1.042	1.017	1.068	0.001
**BMI**^b^	1.105	1.044	1.169	0.001	1.105	1.045	1.169	0.001
**Nitrates**	---	---	---	---	1.882	1.005	3.525	0.048

Models 1 and 2 as described in Table 2

**BMI:** body-mass Index; **CI:** confidence interval

^a^: Hazard ratio estimated by every increase of 1 year.

^b^: Hazard ratio estimated by every increase of 1 kg/m^2^.

Thirty-three patients developed HF or death. This end point was met by 28 patients (6.49%) on PPIs, and by 5 patients (1.81%) not receiving this therapy. There were 16 episodes of HF and 23 deaths, with 6 patients experiencing 2 events. Nine deaths were due to cardiovascular causes (3 of them were sudden death) and 4 were due to malignancies. Infection, renal failure, bowel ischaemia, GI bleeding, and pancreatitis accounted for 1 death each. Five deaths were of unknown cause. Therapy with PPIs was also an independent predictor of this outcome (Table 4).

**Table 4:** Cox proportional hazards model for the incidence of heart failure or death.

	Model 1				Model 2			
	Hazard ratio	95% CI		*P* value	Hazard ratio	95% CI		*P* value
		Lower	Upper			Lower	Upper	
**Atrial fibrillation**	3.531	1.508	8.270	0.004	6.884	2.788	16.999	<0.001
**Diabetes**	0.607	0.416	0.885	0.010	---	---	---	---
**Age**^a^	1.062	1.011	1.115	0.016	---	---	---	---
**CKD-EPI**^b^	0.958	0.933	0.983	0.001	0.951	0.932	0.971	<0.001
**Previous PCI**	1.522	1.040	2.227	0.031	---	---	---	---
**Hypertension**	---	---	---	---	6.443	0.852	48.696	0.071*
**Nitrates**	---	---	---	---	4.554	2.135	9.713	<0.001
**PPIs**	---	---	---	---	5.713	1.628	20.043	0.007
**Peripheral artery disease**	---	---	---	---	0.466	0.250	0.869	0.016
**LDL**	---	---	---	---	0.983	0.966	1.000	0.045

Models 1 and 2 as described in Table 2

**CI:** confidence interval; **CKD-EPI**: glomerular filtration rate assessed as Chronic Kidney Disease Epidemiology Collaboration method; **LDL:** low-density lipoprotein; **PCI:** percutaneous coronary intervention; **PPIs:** proton-pump inhibitors

* Although this P value failed to reach statistical significance, this variable was maintained in the model because the P value calculated with the likelihood ratio method was 0.015

^a^: Hazard ratio estimated by every increase of 1 year.

^b^: Hazard ratio estimated by every increase of 1 ml/min/1.73m^2^

**Fig 1:**
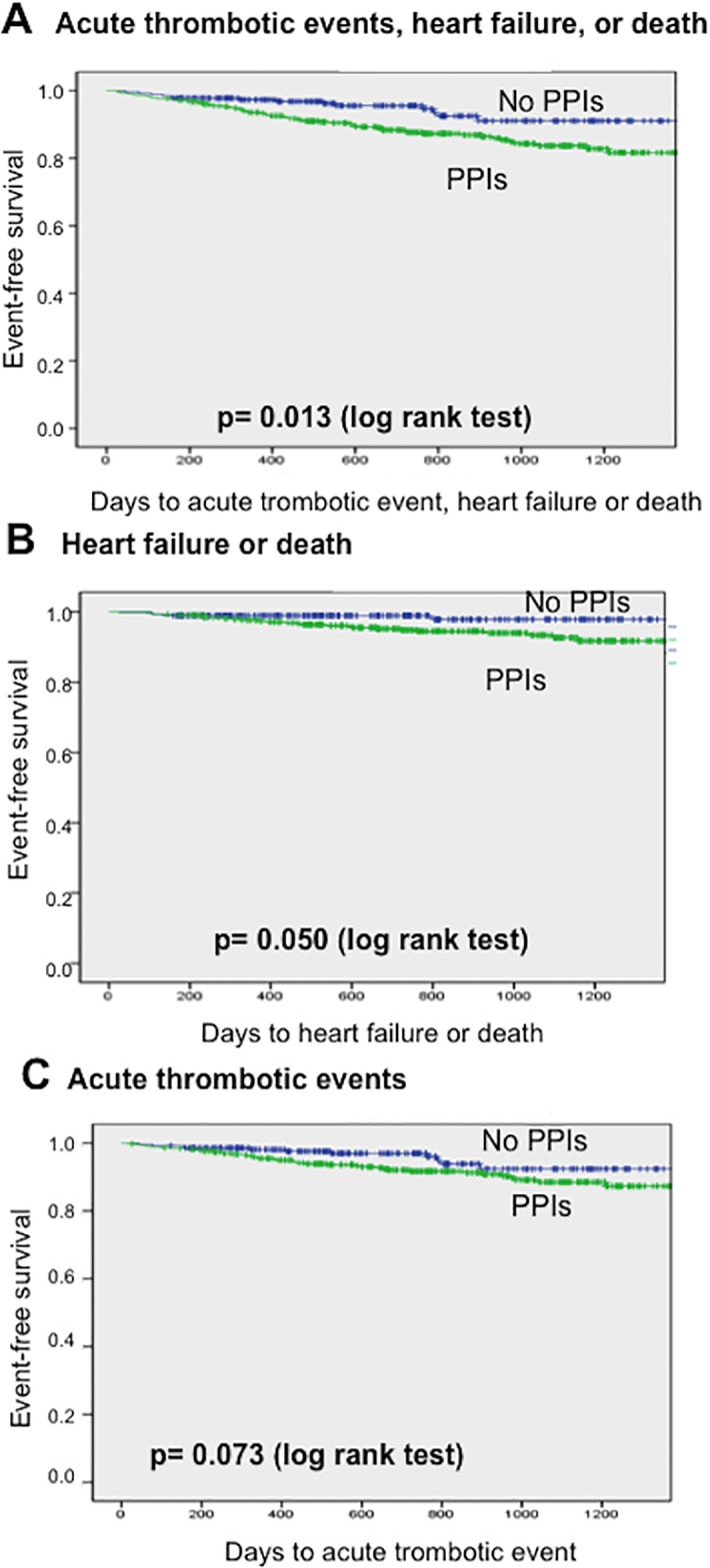
Kaplan-Meier curves showing time to the outcomes in patients with or without PPIs. (A) Time to primary outcome (acute ischaemic events, heart failure or death). (B) Time to heart failure or death. (C) Time to acute ischaemic events.

Kaplan-Meier curves showed that patients on PPIs developed more often the primary outcome (p = 0.013; log-rank test) (Fig 1A) and a borderline *“p”* value (p = 0.050) for the secondary outcome of heart failure or death (Fig 1B).

Finally, we performed a propensity score analysis. In the first step, we included clinical variables, and treatment with PPIs significantly increased the incidence of the primary outcome (HR = 1.912, [95% CI = 1.037–3.523]; p = 0.028). The combined outcome of HF or death showed a result in the limit of statistical significance (HR = 2.921, [95% CI = 0.867–9.840]; p = 0.050). In the second step, we added concomitant drug therapy, showing that PPI treatment increased the incidence of the primary outcome (HR = 1.938, 95% CI = 1.050 to 3.576, p = 0.025), although the combination of HF or death lost the statistical significance (HR = 2.767, 95% CI = 0.817 to 9.373, p = 0.066).

## Discussion

It has been suggested that treatment with PPIs are associated with an increased incidence of cardiovascular events in patients with CAD, mainly in those receiving clopidogrel [14].

In the present study, PPI treatment in patients with CAD was associated with a significant increase in the incidence of HF or death. The distribution of clinical and analytical variables across patients taking PPIs was similar to that of patients not taking these drugs, though with 2 exceptions. First, patients on PPIs were older. This is logical, since age is a risk factor for GI bleeding [17]. Second, patients receiving PPIs had a more frequent history of cerebrovascular events. Although the rate of anticoagulation in these patients was slightly higher than in those not on PPIs, this difference did not reach statistical significance. Moreover, these factors were included in a multivariable analysis, and PPI treatment remained an independent predictor of adverse events. The only exception was the fully adjusted propensity score for the development of HF or death, which lost significance, likely due to the limited number of patients presenting this outcome.

More than 90% of patients were taking aspirin. Chronic treatment with aspirin is a risk factor for GI bleeding regardless of the dose used [18]. Furthermore, two-thirds of the patients were treated also with clopidogrel, and no differences were observed between the PPI and non-PPI groups for this variable. Of interest, no significant interaction was found between clopidogrel use and the association of PPI administration with the primary outcome. Moreover, none of our patients was taking prasugrel or ticagrelor, since at the time this investigation was performed only clopidogrel was available in the participating hospitals.

The present work shows that PPI use is an independent predictor of HF or death. Although there are no previous studies reporting this association, it is known that pantoprazole may exert negative inotropic effects on isolated myocardium from humans and rabbits [19]. This effect was dose-dependent and partially reversible. PPIs decrease gastric acid secretion by blocking the gastric acid pump H^-^/K^+^-adenosine triphosphatase (ATPase). This pump is also present in the myocardium of rats [20]. Inhibition of H^-^/K^+^ ATPase might therefore induce cellular acidosis and a secondary depression in myocardial contractility. However, this was not the mechanism of action of pantoprazole, as no significant changes in intracellular pH could be detected, and all effects occurred at pH 7.3–7.4. Two underlying mechanisms for the pantoprazole-dependent inhibition of contractile force have been described [19]: (1) a decrease in the amplitude of Ca^2+^ transients due to an impaired sarcoplasmic reticulum Ca^2+^ uptake and diminished Ca^2+^ influx via *I*_*Ca L*_, and (2) reduced Ca^2+^ responsiveness of the myofilaments as a consequence of decreased maximal active tension and mildly lower Ca^2+^ sensitivity. Similar results have been obtained with esomeprazole [19] and omeprazole [21], suggesting a class effect.

Although the findings from laboratory studies have been promising, there is controversy regarding the clinical effects of PPIs on myocardial function. Using echocardiography, Schillinger *et al* did not observe any effect of high doses of intravenous pantoprazole on left ventricular ejection fraction or several hemodynamic parameters in healthy volunteers [22]. On the other hand, Tanaka *et al* showed that chronic administration of PPIs in patients with stable angina could be associated with a decrease in ejection fraction and an increase in end-systolic volume index [23].

The increase in mortality associated with PPI use described in the present paper is consistent with previous data. The use of high doses of PPIs has been associated with increased mortality in 491 older patients discharged from acute care hospitals, even when multivariable analysis including predictors of adverse outcomes was carried out [5]. Similarly, PPI use was independently associated with all-cause mortality in 2 cohorts of older patients in long-term care hospitals, acute geriatric wards, and nursing homes [5,24]. Similar results were seen in patients discharged from acute care hospitals [25].

Several potential mechanisms have been suggested to explain the relationship between PPIs and the risk of death [26]. First, the suppression of gastric acidity and the alteration in gut bacterial flora may be the cause of the higher prevalence of *Clostridium difficile* infections and community-acquired pneumonia described in long-term PPI users [5,27,28]. Second, PPI use may cause vitamin B_12_ deficiency, thus leading to a poor nutritional status [29]. In fact, abolishing acid production may interfere with the absorption of nutrients, enhancing the risk of malnutrition [30]. Third, the U.S. Food and Drug Administration reported in 2011 that prescription of PPI drugs for prolonged periods could cause hypomagnesaemia. The mechanism responsible is unknown but may be associated with changes in intestinal absorption of magnesium. Hypomagnesaemia also produces impaired parathyroid hormone secretion, which may lead to hypocalcaemia. Furthermore, as the intracellular concentration of magnesium is involved in the regulation of potassium channels, low magnesium levels may lead to urinary potassium excretion and subsequent hypokalaemia [31,32]. Then, these electrolyte disturbances could cause both supraventricular and ventricular arrhythmias with cardiac arrest or death [33,34]. Nevertheless, future investigations with larger populations are needed to at least confirm a relationship between PPI use and the occurrence of sudden death. Finally, a higher risk of bone fractures has been described in older people taking PPIs [35]. Although the cause of death was available in our patients, the number of specific events for each cause was too small to obtain a reliable estimate of the association between the use of PPIs and each specific cause of death.

In contrast to our data, Oudit *et al* found that PPI use was not associated with all-cause mortality in a cohort of 22,107 patients over age 65 with HF [36]. However, they had a different profile from that of our study, including older age, more comorbidities, and higher mortality.

Use of PPIs was not an independent predictor of acute ischaemic events. Pharmacokinetic and pharmacodynamic studies suggest that use of PPIs may reduce the antiplatelet effects of clopidogrel. The strongest evidence for such an interaction has been found between omeprazole and clopidogrel [37,38]. Ho *et al* reported an increased incidence of hospitalisation for acute coronary syndromes or death in patients with acute coronary syndrome receiving clopidogrel and PPIs [14]. However, recent retrospective studies including propensity score matching have not confirmed these data [39]. In addition, the only randomised double-blinded trial available compared omeprazole and placebo in 3,873 patients with indication for dual antiplatelet therapy, and did not find more adverse cardiovascular events in the PPI group [3]. However, this trial was terminated before schedule and the number of events was lower than expected. In our study there were no differences in the use of aspirin and clopidogrel in PPIs users *vs*. non-users. What is more, treatment with clopidogrel did not affect the association between therapy with PPIs and the incidence of the primary outcome. It therefore seems reasonable to conclude that PPIs had no effect on the incidence of acute ischaemic events and that clopidogrel use did not affect the association observed with the primary outcome.

Finally, we were unable to explore whether adverse prognosis was associated with all PPIs or only with omeprazol, given that the number of patients with other PPIs was low. Similarly, our study did not allow us to analyse whether the adverse effect of PPIs was evident only when comparing these patients to those taking antiH_2_ or, alternatively, if this effect was also present when comparing PPI patients with those receiving no gastric protectors. The reason for this is that, in accordance with the current clinical practice, the number of patients without any gastric protector was very low.

This work has certain limitations. First, this is a non-randomised study with some significant differences at baseline between patients receiving PPI and those who did not. Although we included these variables in the multivariate and propensity score analysis, the small sample size may have limited the statistical power. Second, excluding patients with clinical instability in the first days after the index event may have introduced a bias, as these patients would probably have evidenced a worse prognosis. Nevertheless, only 9 percent of cases were excluded for this reason and the remaining exclusion criteria were designed to prevent the inclusion of cases that could have yielded confounding information, such as patients with major additional disorders. Third, the number of total events was small, thus limiting the strength of the statistical analysis. Fourth, there were few deaths, making it impossible to test whether there was an association between use of PPIs and some specific cause of death. Fifth, the study quality is modest, as more than 50% of patients discharged from the hospitals with a diagnosis of NSTEACS or STEMI were not included in the study based on the exclusion criteria described in the Methods section. Finally, adherence to medication regimens during follow-up was not addressed.

## Conclusions

In conclusion, our results suggest an association between use of PPIs and the incidence of death or HF but do not point to an association with incidence of acute ischaemic events. More studies are needed to confirm these data.

## Supporting Information

S1 File The database of the data availability. (XLSX)
